# Healthy Eating Index Scores, Body Mass Index and Abdominal Obesity Among Asian Americans: NHANES 2011–2018

**DOI:** 10.3389/fepid.2022.879264

**Published:** 2022-04-29

**Authors:** Deena B. Thomas, Tashara M. Leak

**Affiliations:** Division of Nutritional Sciences, Cornell University, Ithaca, NY, United States

**Keywords:** Healthy Eating Index (HEI) 2015, body mass index, waist circumference (From: MeSH), Asian Americans, obesity

## Abstract

**Objective:**

Obesity rates are increasing among Asian Americans so, the objective of this study was to examine associations between Healthy Eating Index scores (a measure of adherence to the Dietary Guidelines for Americans (DGAs)) and body mass index (BMI) and waist circumference (measure of abdominal obesity) among Asian American adults.

**Methods:**

Included in these analyses were Asian Americans ≥20 years from the National Health and Nutrition Examination Survey (2011–18) who had: (1) two 24 h diet recalls to calculate HEI and HEI subcomponent scores, (2) height and weight data to calculate BMI overweight ≥25 kg/m2 and obese ≥ 30kg/m (2), and (3) waist circumference data (high risk ≥ 80 cm for women and ≥ 90 cm for men; *n* = 1024 women and *n* = 904 men). Multinomial logistic regression models were run with BMI as the outcome and logistic regression models were run with waist circumference as the outcome, controlling for age, income, marital status, education, and physical activity.

**Results:**

Among women, a diet with greater adherence to the DGAs for total fruit, fatty acids and total protein was associated with lower likelihood of developing obesity, but a diet with greater adherence to the DGAs in terms of whole grains and sodium (i.e., lower sodium intake) was associated with a higher likelihood of obesity. Among men, greater adherence to the DGAs for fatty acids was associated with a lower likelihood of obesity but greater adherence to the DGAs in terms of whole grains and sodium (i.e., lower sodium intake) was associated with a greater likelihood of obesity.

**Conclusions:**

Future research is needed to examine associations between consumption of specific foods and beverages and how they are associated with BMI and waist circumference among Asian American adults. Furthermore, there is a need culturally relevant obesity prevention interventions for Asian American adults, especially interventions that take into consideration cultural food norms of specific Asian American subgroups.

## Introduction

The number of Asian Americans in the United States (U.S.) increased from 10.5 million to 18.9 million (81% growth) between 2000 and 2019 (1), and obesity is increasingly becoming a concern for this population (2). Between 2011 and 2018, the prevalence of obesity significantly increased from 21 to 33% among Asian American adults. (2) Additionally, Liu et al. (2) analyzed National Health and Nutrition Examination Survey (NHANES) data from 2011–2018 and found that the percent of Asian American women and men combined experiencing abdominal obesity (waist circumference >80 cm for women and >90 cm for men) increased from 22 to 30%. (2) Despite this growing public health issue, little is known about risk factors associated with obesity among the Asian American population.

Diet is a modifiable risk factor for obesity, but little is known about the diet quality of Asian Americans. Diet quality is broadly defined as a dietary pattern or an indicator of variety across key food groups relative to those recommended in dietary guidelines (3). The Healthy Eating Index (HEI) is one measure of diet quality, with scores ranging from 0–100, and higher scores indicate greater adherence to the Dietary Guidelines for Americans (DGAs). Previous studies have reported associations between HEI scores and obesity measures in other populations: higher HEI scores (i.e., greater adherence to the DGAs) have been correlated with lower body mass index (BMI) among European American and “other” American women and men (4), among French women and men (5), and among Non-Hispanic White, Non-Hispanic Black and Hispanic American women and men (6). Additionally, higher HEI scores have been correlated with lower waist circumference in Non-Hispanic White, Non-Hispanic Black and Hispanic American women and men (7). There is, however, very little to no data examining the association between diet quality and obesity among Asian Americans.

The overall aim of the present study is to report on associations between overall diet quality and BMI, as well as waist circumference, among Asian American adults 20 years and older using NHANES data from 2011–2018. The results of the study can be used to inform obesity prevention interventions for Asian Americans.

## Methods

### Study Sample

NHANES is a multi-year, nationally representative survey designed and administered by the National Center for Health Statistics (NCHS) within the Centers for Disease Control and Prevention (CDC) to capture information on nutrition and health in the U.S. Data is collected from civilian and non-institutionalized individuals in 2 year cycles using a complex, multistage probability sampling method to recruit participants. Data were collected during a household interview (demographics, socioeconomic status, health, disease history) and during a clinical exam at the Mobile Examination Center (MEC). At the MEC, anthropometric data and 24 h dietary recall data were collected. A second 24 h dietary recall was collected via telephone 3–10 days later.

For the current study, data from four cycles (2011–2012, 2013–2014, 2015–2016, and 2017–2018) were used to conduct secondary data analysis on men and women who identified as Asian American and were 20 years old or older. Of the 1,540 Asian American women in the sample, 572 were excluded for not having two reliable 24 h dietary recall data and 29 were excluded because they were pregnant, leaving 939 women. For the BMI analysis, of the 939 women, 5 were excluded for missing BMI data and 40 were excluded for being underweight, leaving a sample of 894. The underweight category was not merged into the normal weight category for the final analysis because doing so changed the reported associations. For the waist circumference analysis, of the 939 women who had reliable dietary data, and were not pregnant, 30 were excluded for missing data on waist circumference, leaving 909 women in the final sample. Among the 1,422 men who were Asian American and 20 or older, 493 were excluded for not having two reliable 24 h dietary recall data, leaving 929 men. For the BMI analysis, of the 929 men, 5 were excluded for missing BMI data and 20 were excluded for being underweight. The final sample for the BMI analysis was 904. For the waist circumference analysis, of the 924 who had reliable dietary data, 13 men were excluded for missing data on waist circumference, leaving a final sample of 916 men.

### Measures

The following demographic data were included in the analyses: age (20–29, 30–39, 40–49, 50–59, 60–69, and 70+ years old), sex (female or male), marital status (Married or Partnered, Widowed, Divorced or Separated and Never Married), education (College Graduate or Above, Some College or AA degree or High School or less) and physical activity level (PAL) on a typical day (Low=no vigorous or moderate recreational activity, Moderate= either vigorous or moderate recreational activity, and High= both vigorous and moderate recreational activity). The NHANES physical activity questionnaire is based on the Global Physical Activity Questionnaire and moderate physical activity is defined as any moderate-intensity activity like brisk walking or swimming that leads to a small increase in breathing or heart rate for at least 10 min continuously. Vigorous physical is defined as any vigorous-intensity activity like running or basketball that causes large increases in breathing or heart rate for 10 min continuously. Birthplace was also included in the analyses and it was defined as either being born in the U.S. or outside of the U.S.

Dietary intake data were obtained through two 24 h dietary recalls, which were averaged to calculate HEI-2015 total score [0–100] and the 13 HEI subcomponent scores. A higher HEI total score and higher subcomponent scores are indicative of greater adherence to the 2020–2025 DGAs. The 13 HEI subcomponent scores either fall into the adequate consumption category or moderate consumption category. The nine HEI subcomponents that are recommended for adequate consumption include: total fruits, whole fruits, total vegetables, greens and beans, total protein foods and seafood and plant proteins whole grains, dairy, fatty acids. For example, the HEI subcomponent score range for total fruits is 0–5 and individuals who adhere to the DGAs for total fruit will receive an HEI subcomponent score of 5. The four HEI subcomponents that are recommended in moderation include: refined grains, sodium, added sugars and saturated fats. For example, the HEI subcomponent score range for sodium is 0–10 and individuals who adhere the to the DGAs for sodium will receive an HEI subcomponent score of 10 (i.e., people who consume less sodium will have a higher HEI subcomponent score).

Height and weight measurements were used to calculate BMI by dividing weight (kilograms) by height (meters^2^). These values were used to determine weight status: underweight (BMI: <18.5), normal (BMI: 18.5–24.9), overweight (BMI: 25.0–29.9) and obese (BMI > 30.0) (CDC, 2021). Waist circumference measurements were collected to examine abdominal obesity, and high risk for abdominal obesity is a waist circumference ≥ 80 cm for women and ≥ 90 cm for men (8).

### Data Analysis

Summary and univariate statistics were generated to examine the distribution of HEI scores, BMI, waist circumference, and covariates. Differences in mean BMI and waist circumference across levels of categorical variables was tested using ANOVA. Multinomial regression models were run to assess the relationship between HEI (total and 13 subcomponent scores) and BMI for women and men; the relative risk ratios (RRR) which are the exponentiated coefficients, were reported. Logistic regression models were run to assess the relationship between HEI (total and 13 subcomponent scores) and waist circumference for women and for men; the odds ratios (OR), which are the exponentiated coefficients, were reported. All models were adjusted for age, marital status, education, birthplace, and PAL. All statistical analyses were completed on STATA (STATA/MP 16.1 for Windows).

## Results

### Descriptive Measures

The final analytic sample included 1,825 Asian American adults (≥20 years), of whom 909 were women (49.81%) and 916 were men (50.19%). Participants ranged in age from 20–80 years. Most were married or living with someone (70.79%), had a college degree or above (60.88%). Regarding physical activity, 38.68% of the population engaged in low physical activity, while only 20.82% engaged in high physical activity (Table 1). Differences in mean waist circumference by sex (*p* = 0.001) and marital status (*p* = 0.020) and differences in BMI by sex (*p* < 0.0001) and marital status (*p* = 0.031) were detected (data not shown).

**Table 1:** Descriptive characteristics of 1,825 Asian American adults (≥20 years), NHANES 2011–2018.

	**Unweighted N**	**Weighted %**
**Sex**		
Female	909	50.19
Male	916	49.81
**Age (years)**		
20–29 years	344	18.85
30–39 years	377	20.66
40–49 years	359	19.67
50–59 years	338	18.52
60–69 years	257	14.08
70+ years	150	8.22
**Marital status**		
Partnered	1.292	70.79
Not partnered	173	9.48
Never married	360	19.73
**Highest education**		
College graduate or above	1,111	60.88
Some college or AA degree	345	18.90
High school or less	369	20.22
**Physical activity level**		
High	380	20.82
Moderate	739	40.49
Low	706	38.68
	**Weighted mean**	**SE**
Waist circumference (cm)	88.71	0.42
Body mass index (kg/m^2^)	25.21	0.16
**HEI component scores**		
Total vegetables (0–5)	3.78	0.05
Greens and beans (0–5)	3.01	0.09
Total fruit (0–5)	2.95	0.06
Whole fruit (0–5)	3.31	0.06
Whole grain (0–10)	3.84	0.12
Total diary (0–10)	4.17	0.11
Total protein (0–5)	4.61	0.04
Seafood and plant protein (0–5)	3.76	0.07
Fatty acid (0–10)	6.47	0.11
Sodium (0–10)	2.90	0.09
Refined grain (0–10)	5.33	0.10
Saturated fat (0–10)	7.68	0.10
Added sugar (0–10)	8.54	0.08
Total HEI score (0–100)	60.35	0.49

### HEI and BMI

Among Asian American women, there were no significant associations between total HEI score and BMI, but significant associations were detected between HEI subcomponent scores in the adequacy category and BMI. As total proteins and fatty acids HEI subcomponent scores increased the risk of having an overweight vs. normal BMI was lower (RRR_totalproteins_ = 0.80, *p* = 0.02, 95% CI: 0.65,0/97; RRR_fattyacids_ = 0.94, *p* = 0.04, 95% CI: 0.88,1.00). As total protein, and total fruits HEI subcomponent scores increased, the risk of having an obese vs. normal BMI was lower (RRR_totalproteins_ = 0.65, *p* = 0.0005, 95% CI: 0.52,0.82; RRR_totalfruits_ = 0.81, *p* = 0.006, 95% CI: 0.70,0.94) (Table 2). As whole grain and dairy HEI subcomponent scores increased, the risk for having an overweight vs. normal BMI was higher (RRR_wholegrains_ = 1.09, *p* = 0.002, 95% CI: 1.03,1.15; RRR_dairy_ = 1.11, *p* = 0.0004, 95% CI: 1.05,1.17) and, with whole grains, the risk for having an obese vs. normal BMI was higher (RRR_whole grains_= 1.10, p=0.03, 95% CI: 1.01,1.20) (Table 2).

**Table 2:** Associations between healthy eating index scores and body mass index among Asian American women (*N* = 894) and men (*N* = 904) 20 years and older, NHANES 2011–2018.

	**Women**						**Men**					
**HEI**	**Overweight vs. Normal**			**Obese vs. Normal**			**Overweight vs. Normal**			**Obese vs. Normal**		
	**RRR**	**95%** **CI**	**p**	**RRR**	**95% CI**	**p**	**RRR**	**95%** **CI**	**p**	**RRR**	**95% CI**	**p**
Total vegetables	1.00	0.83, 1.21	0.97	0.92	0.78, 1.08	0.30	1.05	0.90, 1.12	0.55	0.93	0.77, 1.12	0.45
Greens and beans	1.01	0.91, 1.13	0.82	0.91	0.83, 1.00	0.05	1.02	0.91, 1.14	0.74	0.98	0.87, 1.11	0.77
Total fruits	0.95	0.86, 1.05	0.33	0.81	0.70, 0.94	0.006^*^	1.04	0.94, 1.15	0.47	1.00	0.86, 1.18	0.98
Whole fruits	0.98	0.89, 1.07	0.63	0.87	0.75, 1.01	0.07	1.03	0.94, 1.12	0.55	1.00	0.86, 1.16	0.97
Whole grains	1.09	1.03, 1.15	0.002^*^	1.10	1.01, 1.20	0.03	1.04	0.99, 1.09	0.14	1.04	0.95, 1.14	0.36
Total dairy	1.11	1.05, 1.17	0.0004^*^	1.05	0.97, 1.13	0.24	0.97	0.91, 1.03	0.31	1.03	0.93, 1.14	0.60
Total protein foods	0.80	0.65, 0.97	0.02^*^	0.65	0.52, 0.82	0.0005^*^	0.93	0.79, 1.09	0.34	1.25	0.96, 1.63	0.09
Seafood and plant proteins	0.96	0.86, 1.07	0.46	0.90	0.80, 1.01	0.06	1.02	0.91, 1.15	0.69	0.95	0.81, 1.11	0.50
Fatty acids	0.94	0.88, 1.00	0.04^*^	0.93	0.86, 1.01	0.07	1.00	0.94, 1.05	0.80	0.94	0.85, 1.04	0.20
Sodium	1.10	1.03, 1.17	0.007^*^	1.11	1.02, 1.20	0.01^*^	1.09	1.02, 1.16	0.02^*^	1.06	0.95, 1.18	0.30
Refined grains	0.99	0.94, 1.04	0.64	0.94	0.87, 1.03	0.17	1.03	0.98, 1.07	0.21	1.05	0.95, 1.17	0.36
Saturated fat	0.90	0.83, 0.97	0.007^*^	0.87	0.80, 0.95	0.002^*^	1.00	0.93, 1.08	0.94	0.96	0.86, 1.08	0.50
Added sugar	1.00	0.90, 1.11	0.94	1.02	0.88, 1.17	0.84	1.01	0.91, 1.12	0.81	0.98	0.84, 1.14	0.77
Total HEI score	1.01	0.99, 1.02	0.44	0.99	0.96, 1.01	0.22	1.01	0.99, 1.03	0.22	1.00	0.98, 1.03	0.79

* *p < 0.05*.

*HEI, Healthy Eating Index; RRR, Relative Risk Ratio; 95% CI, 95% Confidence Interval; p, p-value*.

*Multinomial Logistic Regression Models were adjusted for age, birthplace, marital status, education and physical activity level*.

Regarding HEI subcomponents recommended in moderation, a few associations with BMI were observed among Asian American women. As the saturated fat HEI subcomponent scores increased (i.e., saturated fat intake decreased), the risk for having an overweight vs. normal BMI was lower (RRR_saturatedfat_ = 0.90, *p* = 0.007, 95% CI: 0.83,0.97), and the risk for having an obese vs. normal BMI was lower (RRR_saturatedfat_ = 0.87, *p* = 0.002, 95% CI: 0.80, 0.95). As the sodium HEI subcomponent score increased (i.e., sodium intake decreased), the risk for having an overweight vs. normal BMI was higher (RRR_sodium_ = 1.10, *p* = 0.007, 95% CI: 1.03,1.17) and the risk for having an obese vs. normal BMI was higher (RRR_sodium_ = 1.11, *p* = 0.01, 95% CI: 1.02, 1.20) (Table 2).

Regarding Asian American men, no significant association was detected between total HEI score and BMI, but as the sodium HEI subcomponent score increased (i.e., sodium intake decreased), the risk for having an overweight vs. normal BMI was higher (RRR_sodium_ = 1.09, *p* = 0.02, 95% CI:1.02, 1.16) (Table 2).

### HEI and Abdominal Obesity

Among Asian American women, no significant associations were detected between total HEI score and abdominal obesity but several significant associations were observed between HEI subcomponent scores. Higher HEI subcomponent scores for total fruits and total protein foods were associated with lower odds of having a waist circumference above 80 cm vs. below 80 cm among Asian American women (OR_totalfruits_ = 0.87, *p* = 0.01, 95% CI:0.77, 0.97; OR_totalprotein_ =0.81, *p* = 0.03, 95% CI: 0.67, 0.98) (Table 3). For every 1-point increase in the HEI subcomponent score for total fruits, there was 13.5% lower odds of having a waist circumference above 80 cm vs. below 80 cm. Similarly, for every1-point increase in the HEI subcomponent score for total protein foods, there was 19.3% lower odds of having a waist circumference above 80 cm vs. below 80 cm. On the other hand, as the HEI subcomponent score for whole grains increased, of having a waist circumference above 80 cm vs. below 80 cm was higher (OR_wholegrains_ = 1.06, *p* = 0.04, 95% CI: 1.00,1.12) (Table 3).

**Table 3:** Associations between healthy eating index scores and waist circumference among Asian American women (*N* = 909) and men (*N* = 916) 20 years and older, NHANES 2011–2018.

**HEI**	**Women**			**Men**		
	**OR**	**95% CI**	**p**	**OR**	**95% CI**	**p**
Total vegetables	0.91	0.80, 1.03	0.14	1.04	0.92, 1.18	0.53
Greens and beans	0.99	0.91, 1.09	0.87	0.97	0.88, 1.06	0.46
Total fruits	0.87	0.77, 0.97	0.013^*^	1.01	0.93, 1.10	0.83
Whole fruits	0.91	0.82, 1.01	0.09	0.98	0.91, 1.07	0.69
Whole grains	1.06	1.00, 1.12	0.04^*^	1.06	1.02, 1.10	0.008^*^
Total dairy	1.03	0.97, 1.10	0.34	1.03	0.97, 1.08	0.36
Total protein foods	0.81	0.67, 0.98	0.03^*^	0.86	0.73, 1.01	0.06
Seafood and plant proteins	1.11	1.00, 1.23	0.06	0.91	0.82, 1.00	0.05
Fatty acids	0.97	0.91, 1.02	0.22	0.95	0.91, 1.00	0.05
Sodium	1.09	1.02, 1.16	0.009^*^	1.11	1.04, 1.17	0.001^*^
Refined grains	0.96	0.91, 1.01	0.11	1.04	0.99, 1.09	0.15
Saturated fat	0.93	0.86, 1.02	0.10	0.95	0.88, 1.02	0.15
Added sugar	1.00	0.91, 1.11	0.94	0.96	0.87, 1.07	0.47
Total HEI score	1.00	0.99, 1.01	0.74	1.01	0.99, 1.02	0.58

* *p < 0.05*.

*HEI, Healthy Eating Index; OR, Odds Ratio; 95% CI, 95% Confidence Interval; p, p–value*.

*Logistic regression models were adjusted for age, birthplace, marital status, education and physical activity level*.

Among Asian American men, no significant association was detected between the HEI total score and abdominal obesity, but HEI subcomponent scores for sodium and whole grains were associated with abdominal obesity. As the HEI subcomponent score for sodium increased the odds of having a waist circumference above 90 cm vs. below 90 cm was higher (OR_sodium_ = 1.11, *p* = 0.001, 95% CI: 1.04,1.17). As the HEI subcomponent score for whole grains increased the odds of having a waist circumference above 90 cm vs. below 90 cm was higher (OR_wholegrains_ = 1.06, *p* = 0.008, 95% CI: 1.02,1.10) (Table 3).

As the HEI subcomponent score for sodium increased (i.e., sodium intake decreased) the odds for of having a waist circumference above the cut-off vs. below the cut-off was higher among women (OR_sodium_ =1.09, *p* = 0.009, 95% CI: 1.02, 1.16) and men (OR_sodium_ = 1.11, *p* = 0.001, 95% CI: 1.04,1.17) (Table 3).

## Discussion

The present study reports how diet is associated with obesity among Asian Americans 20 years and older and it identifies dietary factors associated with risk of obesity. Several dietary components were associated with lower BMI and waist circumference while several were associated with an overweight or obese BMI and higher waist. Among women, a diet with greater adherence to the DGAs for total fruit, fatty acids and total protein is associated with lower likelihood of developing obesity, but a diet with greater adherence to the DGAs in terms of whole grains and sodium (i.e., lower sodium intake) is associated with a higher likelihood of obesity. Among men, greater adherence to the DGAs for fatty acids is associated with a lower likelihood of obesity but greater adherence to the DGAs in terms of whole grains and sodium (i.e., lower sodium intake) is associated with a greater likelihood of obesity.

The inverse relationship we observed between fruit intake and obesity (BMI and waist circumference) among Asian American women has been reported among other Asian Americans and Canadian adults. For example, higher intake of fruit is associated with lower BMI among Asian Americans (9), Filipino Americans (10), and Canadian adults (11). Similarly, lower waist circumference is associated with higher fruit intake among adults in the United States and other countries (12). One possible explanation for these associations is that fruit is typically high in dietary fiber and is associated with extended periods of satiety and lower total energy intake which may lead to less weight gain (13). It is unclear why this association is only seen in women, and further analysis is needed to compare the quantity of fruit eaten by Asian American women vs. men and whether differences in quantity has an impact on obesity.

Interestingly, whole grain intake in our study was positively associated with higher risk for obesity (BMI and waist circumference) among Asian American women and men, contrary to what other researchers have found. O'Neil et al. (14) analyzed NHANES 1999–2004 data and found that U.S adults (all races/ethnicities combined) who consumed more whole grains had a lower BMI and waist circumference (14). Whole grains are a central part of Asian American cuisine and the practice is to consume large amounts of carbohydrates to feel full (15, 16). This, in turn, leads to higher caloric intake and higher risk for obesity. South Asians consume whole grains such as sorghum and millet (17) and East Asians consume whole grains such as black rice, brown rice, and soba noodles (18). It is important to acknowledge that whole grain intake is encouraged, especially since consumption is shown to improve glycemic measures and reduce risk for type 2 diabetes, and obesity-related chronic disease. Research examining the relationship between whole grain consumption and obesity may depend on whether the person is metabolically healthy or not. For example, in a study of U.S. women of multiple race/ethnicities, whole grain intake was higher among those who were metabolically-healthy-obese vs. those who were metabolically-abnormal-obesity (MAO) and it is possible that, in our population, we had a high number of metabolically obese individuals (19).

As HEI subcomponent scores sodium increased (i.e., lower sodium intake) the risk of obesity (BMI and waist circumference) was higher among Asian American women and men, contrary to findings in other studies. Jiang et al. (20) reported that among U.S. adults (all races/ethnicities combined) sodium intake and BMI, as well as waist circumference are positively associated. Also, a study examining associations between diet quality and central adiposity among Mexican Americans found that higher HEI subcomponent scores for sodium (i.e., lower sodium intake) were associated with a smaller waist circumference (21). Among Asian Americans, soups, rice, and yeast breads are the top three food sources of sodium, whereas, among Non-Hispanic Whites, Non-Hispanic Blacks and Hispanics more energy dense foods such as cold cuts, cured meats, meat dishes are the top three food sources of sodium (22). Thus, among Asian Americans, higher salt intake may be a proxy for a diet that is high in fruits and vegetables and low in fat which lowers the risk for obesity and, comparatively lower salt intake maybe a proxy for a more Western dietary pattern, which increases the risk for obesity.

Higher HEI subcomponent scores for fatty acids were associated with lower risk of obesity among Asian American women and men. The HEI fatty acid subcomponent score increases as the ratio of mono- and poly- unsaturated fatty acids to saturated fatty acids (SFAs) increases. So, a higher HEI component score is indicative of lower SFA intake, and the positive effects on BMI when SFAs in the diet are replaced have been reported elsewhere (23). Policy and interventions should encourage continued consumption of foods with unsaturated fatty fats among Asian Americans.

Lastly, protein intake was inversely associated with obesity among Asian American women and findings have been mixed in comparison to other studies. In a population of South Korean adults, inverse associations were reported between protein intake and BMI, modulated by protein quantity such that at lower quantities, protein is inversely associated with BMI and waist circumference and at higher quantities, it is associated with higher BMI and waist circumference (24). In studies of U.S. adults of mixed race/ethnicities, however, protein intake is associated with increased BMI and waist circumference (4, 25). This could be due to a high correlation between protein and saturated fat intake, and the fat intake could be driver of higher BMI and waist circumference. Among Asian Americans, further analysis on protein sources, quantity and fat content is needed to better understand the relationship between protein intake and obesity.

This study has several limitations and strengths that should be considered. First, NHANES uses a cross-sectional study design so, causation between HEI scores and BMI, and waist circumference cannot be inferred. Second, NHANES data on Asian Americans is aggregated even though Asian Americans hail from 20 countries. Hence, it is not possible to detect inter-group differences in diet quality or its effects on BMI or abdominal adiposity. Despite these limitations, this paper addresses a major gap in literature as we are the first to report on the relationship between diet quality, BMI, and waist circumference among a nationally representative sample of Asian American adults. Obesity is becoming a major public health concern for Asian American adults, yet this population remains understudied and underserved. Most obesity-related studies report on BMI, and a strength of the current study is the inclusion of waist circumference data, a measure of abdominal obesity associated with unhealthy eating patterns, diabetes and cardiovascular diseases among populations in Asia and the Asia Pacific region (8, 26–28).

Future research is needed to examine associations between consumption of specific foods and beverages are associated with BMI and waist circumference among Asian American adults. For example, it is possible that certain whole grain foods are associated with obesity (e.g., whole grains foods that are more energy dense) and these foods may vary across Asian American subgroups (e.g., Chinese, Japanese, Korean, etc.). Furthermore, there is a need culturally relevant obesity prevention interventions for Asian American adults, especially interventions that take into consideration cultural food norms of specific Asian American subgroups.

## Data Availability Statement

Publicly available datasets were analyzed in this study. This data can be found here: https://wwwn.cdc.gov/nchs/nhanes/.

## Ethics Statement

Ethical review and approval was not required for the study on human participants in accordance with the local legislation and institutional requirements. The patients/participants provided their written informed consent to participate in this study.

## Author Contributions

DT and TL contributed to the conception and design of the study. DT organized the database, performed the statistical analysis, and wrote the first draft of the manuscript. Both authors wrote sections of the manuscript, contributed to manuscript revision, and approved the submitted version.

## Conflict of Interest

The authors declare that the research was conducted in the absence of any commercial or financial relationships that could be construed as a potential conflict of interest.

## Publisher's Note

All claims expressed in this article are solely those of the authors and do not necessarily represent those of their affiliated organizations, or those of the publisher, the editors and the reviewers. Any product that may be evaluated in this article, or claim that may be made by its manufacturer, is not guaranteed or endorsed by the publisher.
